# Development of Probiotic Candidate in Combination with Essential Oils from Medicinal Plant and Their Effect on Enteric Pathogens: A Review

**DOI:** 10.1155/2012/457150

**Published:** 2012-07-03

**Authors:** Shipradeep, Sourish Karmakar, Rashmi Sahay Khare, Sumedha Ojha, Kanika Kundu, Subir Kundu

**Affiliations:** ^1^School of Biochemical Engineering, Indian Institute of Technology, Banaras Hindu University, Varanasi 221005, India; ^2^Chemistry Section, MMV, Banaras Hindu University, Varanasi 221005, India

## Abstract

Medicinal plants and probiotics both have very high potential in terms of their antimicrobial activity against antibiotic-resistant enteric pathogens. The probiotics being enteric microorganism do not have any parasitic effect on human beings. They have been an integral part of daily food for centuries. They have been shown to have health beneficiary properties. The probiotics retard the growth of the microorganisms, while essential oil kills them. Combining the effect of medicinal plant extract and probiotics may be a new approach due to their complementary antimicrobial effects and practically no side effects. The synergistic effect of the essential oil and probiotics will be necessarily higher than using them alone as health product.

## 1. Introduction

The plants have been used in Ayurvedic medicines from ancient times. The extracts from these plants have shown potent antimicrobial effect. Recently, much work has been done on extraction of chemicals responsible for the antimicrobial effect from these plant species. It has been reported that the essential oils extracted from these plants have potent activity against microorganisms [1]. However, the studies have shown that these essential oils have very high MIC (minimal inhibitory concentration) against beneficial enteric bacteria known as probiotics [2, 3]. 

Probiotic is the term as per WHO definition denotes “live microbial feed supplement which beneficially affects the host animal by improving its intestinal microbial balance.” As the definition clearly indicates, most of the intestinal bacteria have an important role to play in the digestive system. Earlier, probiotics were given to animals to improve their health, but later much research has been put in the development of the probiotics for human health. The major probiotics that are taken in the diets belongs to the genera of *Lactobacilli* and *Bifidobacteria* [4]. Apart from that, the gut flora predominately has obligate anaerobes that include *Bifidobacteria, Clostridia, Eubacteria, Fusobacteria, Peptococci, Peptostreptococci*, and *Bacteroides*. Only about 1% of these bacteria are facultative anaerobes belonging to the genera of *Lactobacilli, Escherichia coli, Klebsiella, Streptococcus, Staphylococcus*, and* Bacilli*. In the case of newborns, food habits play a major role in the development of enteric flora. The breastfed babies normally have abundance of *Bifidobacteria*, while the others have complex microflora in their enteric system. *Bifidobacterium *sp. can be isolated mostly from the feces of infant milk feed baby. However, in the case of infants fed on normal formula based food products the gut flora is found to be rich in *Enterobacteria, Lactobacilli, Bacteroides, Clostridia*, and *Streptococci.* These gut flora help to digest the milk-based food and offer the primary line of defense against the pathogenic bacteria. The infants have weak but developing immune system [5]. These enteric bacteria help the infantile immune system to fight against pathogenic enteric bacteria by lowering the pH of the gut, rendering it unsuitable for pathogenic bacteria to survive [6]. Even the medical practitioners recommend probiotics-based supplement to both patients suffering from enteric diseases. The most popular probiotics supplements belong to the genera of *Lactobacilli* and *Bifidobacteria. *The recommended dosage of 10^9^–10^10^ CFU is considered a minimum for healthy enteric system [7].

Present review emphasizes on the synergistic antimicrobial effect of essential oil of the Lamiaceae family and probiotics administered together as flavored fermented milk products. The advantage of using such a combination is its beneficial effect with its antimicrobial property. The probiotics can help in improving the gut epithelial conditions while essential oil acts on killing the pathogens present in the human body. 

## 2. Health Benefits of Probiotics

Probiotics, though recently popular, have been an integral part of the human diet for centuries. All the civilizations from ancient times have documented the benefits of curd in the human diet. The lactose-tolerant people are always advised to take curd with their diet. The curd is rich in *Lactobacillus* sp. and *Streptococcus* sp. These microorganisms utilize the lactose present in milk-based food and convert it to lactic acid [8]. The occurrence of flatulence in carbohydrate-intolerant individuals is also observed with fatty acid. The carbohydrates that are not fully digested due to lack of certain enzymes in human being can also be digested with probiotics. These carbohydrates are fermented into short-chain acids such as butyric acid, lactic acid, or acetic acid [9]. These acids are readily utilized in by human cells for ATP metabolism providing energy to the individuals. The lactic acid also helps in protein metabolism by coagulating the protein chunks from meat inside the intestine [10]. Formation of hydrogen peroxide is also prevented by catalases produced from probiotics preventing protein-caused rancidity [11–13]. Hydrolysis of sarcoplasmic protein was also observed with many species of *Lactobacillus* genus [14–16]. Coprecipitation of cholesterol with bile salts at lactic acid-induced lower pH is also observed in in vitro conditions [17]. 

Probiotic microorganisms are also found to be involved in synthesis of vitamins. The probiotics microorganisms are known to synthesize biotin and vitamin K [18]. Apart from that, they are also involved in the ions absorption such as Mg^2+^, Ca^2+^, and Fe^3+^. 

The probiotic microorganisms are also involved in the enhancement of expression of certain pattern recognition receptors. Pattern recognition receptors such as TLRs have active role in wound healing process. The intestinal cells have high need for these receptors for supporting their process of proliferation and differentiation, healing the wounds made due to irregular bowel movement [19]. The short-chain fatty acid produced from carbohydrate metabolism also enhances the process of proliferation and differentiation of gut epithelial cells. 

The probiotics have also a major role to play in prevention of allergies in children [5]. However, the connection of probiotics and immune system regulation is still under investigation. It has been observed that with allergy-prone adults and children, the count of *Lactobacilli* and *Bifidobacteria* is lower. It has been also observed that administration of probiotic strains during prenatal stage can decrease the chance of atopic eczema. In addition, the production of pattern recognition receptors, interleukin, and growth factors from the probiotic microorganisms in gut epithelia also play an important role in prevention of allergies. Therefore, it can be inferred that these microorganisms have direct role in immune system regulation [5]. Apart from that, these microorganisms also play a role in immune response modulation. The probiotic microorganisms interact with the gut-associated lymphoid tissue (GALT) [20]. The probiotics are involved in cytokine synthesis, that plays an important role in immune system regulation. However, due to insufficient clinical trial, administration of probiotics in immunosuppressed individuals is still prohibited. 

It has been also observed from both in vitro and in vivo studies that probiotics may prevent cancer [21]. It has been found that daily intake of fermented milk products substantially decrease the concentration of nitroreductases, azoreductases, and *β*-glucuronidase in the gut. These microbial enzymes are associated with carcinogen production in the gut [22]. *Lactobacillus casei* have also shown an antigenotoxic effect. It prevents inducible DNA damage in the tumor target tissues of gastrointestinal tract of rats.

## 3. Antimicrobial Effect and Mechanism of Action

Probiotics have a known antimicrobial effect. They are very potent against pathogens. There are several proposed mechanisms for the antimicrobial action of the probiotics. Bacteriocins, organic acids, hydrogen peroxide, diacetyl, and other inhibitory chemicals are released by the probiotics [23]. All of these chemicals are known for their potent antimicrobial effects. Bacteriocins are toxic chemicals released by the probiotics, that are highly potent against most of the bacteria. However, the most feasible mode of action seems to be lowering of pH with release of organic acids such as lactic acid [24, 25]. In the limiting condition of available substrates inside the intestine, lowering the pH ensures the survival of acidophilic micro-organisms only. The growth of the pathogens gets inhibited at acidic conditions, slowing the metabolic process in them. Lactobacillus strain GG has been reported to produce inhibitory chemicals, possibly a microcin, that have high activity against pathogenic microorganisms. It has been found effective against *Clostridium spp., Bacteriodes spp., Enterobacteriaceae spp., Staphylococcus spp.,* and *Pseudomonas spp*. in microbiological assays. Lactocidin released by strains of *lactobacillus acidophilus* is found active against *Staphylococcus aureus* and *Pseudomonas aeruginosa* [26]. There has been a study that *Lactobacillus acidophilus* LB release chemicals that are effective against both gram positive and gram negative microorganisms. These chemicals released in the broth were effective against *Staphylococcus aureus*, *Listeria spp.*, *Salmonella typhimurium*, *Shigella flexneri*, *E. coli*, *Klebsiella pneumoniae*, *Bacillus cereus*, *Pseudomonas aeruginosa*, and *Enterobacter *spp. [27]. However, the chemical did not have any inhibitory effect on probiotics strains such as *Lactobacillus* and *Bifidobacterium spp*. This can be explained by the similarity of survival conditions of both these microorganisms. Some of the strains of *Bifidobacterium spp*. have potent activity against *Salmonella typhimurium*. However, not all the strains of *Bifidobacterium* spp. have the activity against *S. typhimurium*. All of the probiotics have higher survivability in low pH conditions. These microorganisms produce acids by breaking the carbohydrate present in the diet. The properties of acid production and acid survivability increase their survivability in the toughest of conditions [28, 29]. The adherence property of the probiotic microorganisms also ensures their longevity in the human guts [30]. However, the probiotic strains have shown an effective potential in inhibiting the adhesion of pathogen such as *E. coli *and *Salmonella enterica* in in vitro conditions [31]. The potential of adhesion inhibition by the probiotics is credited to the mucin production and competitive binding to gut epithelial receptor sites. *Lactobacillus acidophilus* LA1 has high calcium independent adhesive property that inhibits the invasion of enteropathogenic bacteria. Mucins are complex glycoprotein that inhibits the enterobacterial adhesion by protection of intestinal epithelial cell receptors. Both MUC2 and MUC3 produced by *Lactobacillus spp.* are potent examples of Mucins that have adhesion inhibitory activity against enteropathogens.

## 4. Antimicrobial Effects of Essential Oils from Medicinal Plants 

There has been lot of studies in recent year that have established the antimicrobial effect of essential oils of medicinal plants such as plants of the Lamiaceae family [2, 3]. The essential oils predominately present in the leaves of the plant species have a pleasant aroma. They are commonly used in flavor enhancement in food industries, as they are safe for human consumption. These essential oils have been shown to have a bactericidal effect. The plant species of Lamiaceae family have been proven effective against Uropathogen [32].  Table 1 shows the MICs of *Coleus aromaticus* and *Ocimum sanctum* (*Rama Tulasi* and *Shayama Tulasi*) against few known enteric pathogens [32]. The essential oil from the plants of *Carum carvi, Coleus aromaticus, Rama Tulasi, Shyama Tulasi, Citrus aurantium *var. *amara*, *foeniculumvulgare dulce, Illicium verum, Lavandula angustifolia, Mentha arvensis, Mentha x piperita*, and* Trachyspermum copticum* have been shown to be effective against variety of microorganisms. These plants extracts have been found effective against *Bacteroides fragilis, Candida albicans, Clostridium difficile, Clostridium perfringens, Enterococcus faecalis, Escherichia coli, Eubacterium limos, Staphylococcus aureus, Klebsiella oxytoca, Proteus vulgaris, Escherichia coli, Klebsiella pneumoniae, Pseudomonas aeruginosa, Proteus mirabilis*, and *Peptostreptococcus anaerobius* [2]. The MICs against these microorganisms varies from 0.1 to 3%v/v. The MICs of the same plant extracts against probiotic microorganisms such as *Bifidobacterium bifidum, Bifidobacterium longum, Lactobacillus acidophilus*, and *Lactobacillus plantarum *are much higher in magnitude than the pathogens [2]. Therefore, if the dosage of essential oil is low, then it effectively wipes out the pathogens without harming the beneficial probiotics. 

## 5. Proposal on Synergistic Effect of Probiotics and Essential Oil from Plants

The essential oils have high MIC values for probiotics, while it is effective in much lesser concentration against the pathogens. The above phenomenon makes it possible that both probiotics and essential oil can be administered together to cure pathogenic infection in human gut. They both can be combined to form essential oil-flavored fermented milk products such as flavored curd beverages or flavored yogurt. Antibiotics coupled with probiotics are already present in the market, but these medicines mostly face stiff challenge from antibiotic-resistant bacteria. Further frequent use of the antibiotics may lead to the development of antibiotic resistance in the pathogenic microorganisms too. Hence, the strategic use of probiotics may be beneficial to curb the growing phenomenon of antibiotic resistance. Probiotics have antimicrobial properties associated with the production of bacteriocin-like chemicals. However, it mostly arrests the proliferation of the pathogens by lowering the pH in the gut environment. The pathogens do not normally have any mechanism against the action of essential oils. Essential oils are resistant against enzymatic activity of *β*-lactamase produced as a countermeasure against *β*-lactam antibiotics. The use of probiotics lowers the survivability chances of pathogen, while the essential oil in lower dosage ensures their complete killing inside the human digestive tract. The probiotics may also impart its good benefits discussed earlier. Apart from that, the fermented milk product will surely impart benefits in terms of supplying nutrients such as sugar, water, salt, and acid to the human body. Adding essential oil will not only give an aromatic flavor to these fermented milk beverages or products, but also increase their shelf like considerably by preventing the microbial spoilage. The product will act as both probiotic health product and preventive antimicrobial product against enteric pathogens. 

In an independent study, beverages A, B, and C were prepared with probiotic curd (10^9^ CFU/ml) [33] with varying concentration of essential oil of *Coleus aromaticus, Rama Tulasi* and *Shyama Tulasi*, respectively [1]. The beverages A1, A2, and A3 were prepared with essential oil of *Coleus aromaticus*; beverage B1, B2, and B3 with essential oil of *Shyama Tulasi* and beverages C1, C2, and C3 with essential of *Rama Tulasi* in varying concentrations of 1, 2, and 3 *μ*l/ml respectively. These beverages were then grown with common enteric pathogens in equal concentration, measured by count of CFU, in nutrient broth for 24 hours in airtight culture vials at 37°C to simulate the anaerobic condition prevailing in the intestine [34]. The individual vial was tested for the traces of pathogen as seen in Table 2 with − sign indicating the cidal effect of the beverage against the pathogen (no growth of the pathogens), while + sign indicated the growth of the pathogen. The sample beverages were found to be highly effective in inhibiting the growth of the pathogen. The shelf life of the beverages was also found to be significantly higher than normal probiotics [34]. The test results can be interpreted as the beverage's capacity for prevention against enteric pathogens. The use of beverage does not need the stringent FDA regulations, yet it will impart the benefit of preventive diseases. 

## 6. Conclusion 

Probiotics and essential oils both have a great potential in terms of their beneficial effect against microbial gut infection. They also show a synergistic effect that is normally higher than any known drug due to their complementary actions. Since most of these medicinal plants are edible, their extracts as food product do not have any side effects with low dosage. Therefore, these products may be very beneficial for human beings. However, much research is needed to be put into these studies, as drug regulatory authorities still have strong regulations against usage of plant extracts as medicines. 

## Figures and Tables

**Table 1:** MICs of essential oil against known pathogenic microorganisms.

Bacterial strains	*Coleus aromaticus*	*Shyama tulasi *	*Rama tulasi *
*Providencia rettgeri *	~4 *μ*l/mL	~3 *μ*l/mL	~2 *μ*l/mL
*Shigella flexneri *	~1 *μ*l/mL	~5 *μ*l/mL	~5 *μ*l/mL
*Shigella dysentery *	~3 *μ*l/mL	—	~5 *μ*l/mL
*Vibrio parahaemolyticus *	~2 *μ*l/mL	~5 *μ*l/mL	~5 *μ*l/mL
*Salmonella enteritis *	~4 *μ*l/mL	~2 *μ*l/mL	~3 *μ*l/mL
*Salmonella typhi *	~0.5 *μ*l/mL	~2 *μ*l/mL	~2 *μ*l/mL
*Vibrio cholerae *	~2 *μ*l/mL	~2 *μ*l/mL	~1 *μ*l/mL

**Table 2:** Test of the beverages made with mixing different concentration of essential oil of *Coleus aromaticus, Rama tulasi, *and *Shyama tulasi *against common pathogens for 24 hrs at 37^°^C (− sign denotes no pathogen; + sign denotes the presence of pathogen).

S.	Bacteria	Gram	Beverage	Beverage	Beverage	Beverage	Beverage	Beverage	Beverage	Beverage	Beverage
number		+ve/−ve	1	2	3	4	5	6	7	8	9
(1)	*Citrobacter freundii*	−ve	−	−	−	−	−	−	−	−	−
(2)	*Proteus mirabilis*	−ve	+	+	+	−	−	−	+	−	−
(3)	*Klebsiellal pneumoniae*	−ve	+	+	+	+	+	+	+	+	−
(4)	*E. coli* ATCC 25922	−ve	+	−	−	+	+	+	+	−	−
(5)	*Entero. faecalis* ATCC 29912	+ve	−	−	−	−	−	−	−	−	−
(6)	*Salmo. typhi* MTCC 3216	−ve	−	−	−	−	−	−	+	+	−
(7)	*Staph. aureus* ATCC 25923	+ve	+	+	−	+	+	−	+	−	−
(8)	*Salmonella typhimurium*	−ve	+	−	−	−	−	−	−	−	−
(9)	*Salmonella paratyphi*	−ve	+	+	−	+	−	−	+	+	−
(10)	*Vibrio cholerae*	−ve	−	−	−	−	−	−	−	−	−
(11)	*Pseudo. aeru*. ATCC 27853	−ve	−	−	−	−	−	−	−	−	−
(12)	*Proteus vulgaris*	−ve	+	+	−	−	−	−	+	−	−
(13)	*Listeria monocytogenes*	+ve	+	+	−	+	+	−	+	−	−
(14)	*Shigella flexneri*	−ve	−	−	−	+	−	−	+	+	−
(15)	*Helicobacter pylori*	−ve	−	−	−	−	−	−	−	−	−
(16)	*Strptococcus heamophila*	+ve	+	−	−	−	−	−	−	−	−
